# Supplementary material for: Emergence of supercoiling-mediated regulatory networks through the evolution of bacterial chromosome organization
Source: PLoS Comput Biol. 2025 Sep 29;21(9):e1013482. doi: 10.1371/journal.pcbi.1013482 (PMC12503335; doi:10.1371/journal.pcbi.1013482)
Supplement: S1 Text — (PDF) [file pcbi.1013482.s001.pdf]

S1 Supporting Text

List of Supplementary Figures

A Average fitness during evolution, with decreasing environmental supercoiling shifts 2

B Evolution of the number of activated genes in each environment, with decreasing environmental supercoiling shifts 2

C Average gene expression as a function of background supercoiling, with an absolute environmental supercoiling shift of 0.001 3

D Average gene expression as a function of background supercoiling, with an absolute environmental supercoiling shift of 0.0001 3

E Average fitness during evolution with a smaller gene interaction distance 4

F Evolution of the number of activated genes in each environment with a smaller gene interaction distance 4

G Average fitness during evolution with constant $N\lambda$ but different N and λ 5

H Evolution of the number of activated genes in each environment with constant $N\lambda$ but different N and λ 5

Smaller Environmental Supercoiling Perturbations

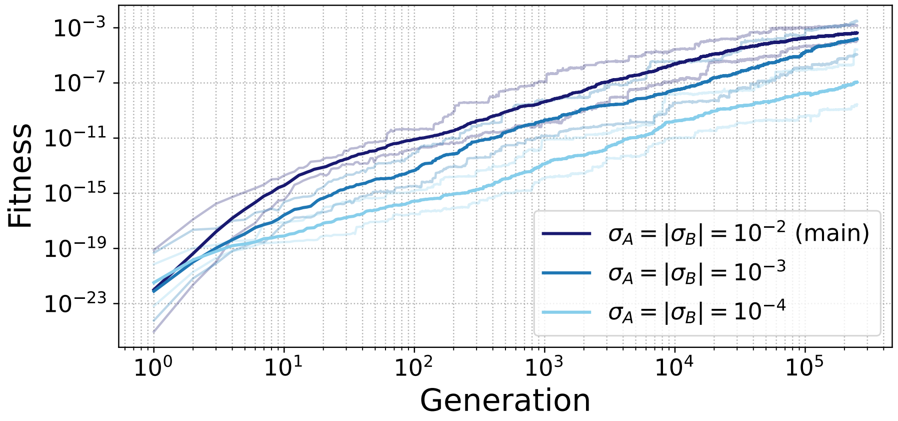

Figure A: Average fitness during evolution, with environmental shifts in supercoiling logarithmically decreasing in absolute value: $\sigma_A = 10^{-2}$ and $\sigma_B = -10^{-2}$ (main run), $\sigma_A = 10^{-3}$ and $\sigma_B = -10^{-3}$ (10 times smaller than in the main run) and $\sigma_A = 10^{-4}$ and $\sigma_B = -10^{-4}$ (100 times smaller than the main run). Lighter lines represent the first and last decile of the data.

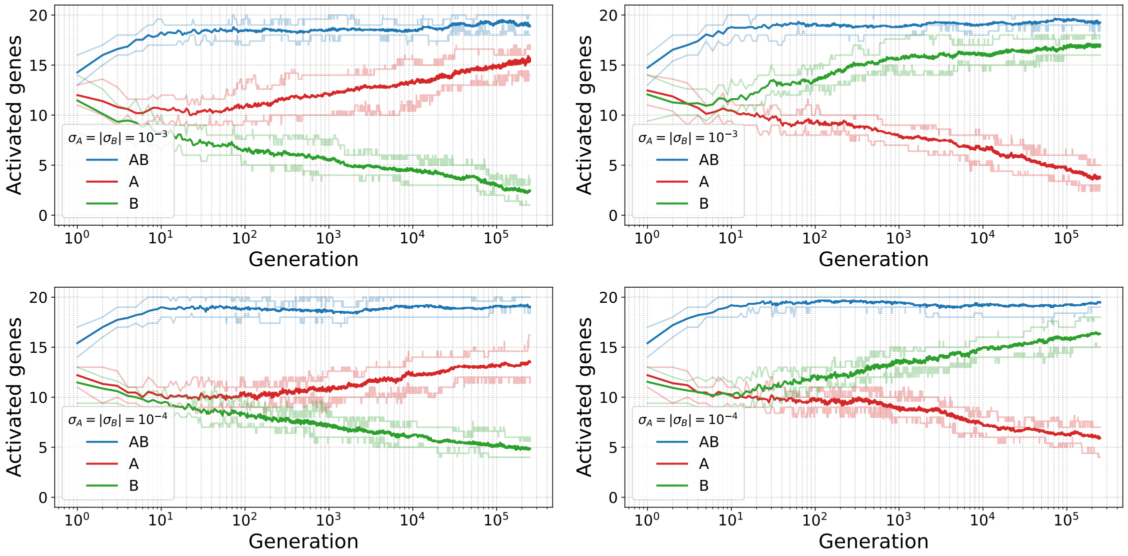

Figure B: Evolution of the number of activated genes in environment A (left) and environment B (right), with environmental supercoiling shifts $\sigma_A = 10^{-3}$ and $\sigma_B = -10^{-3}$ (top) and $\sigma_A = 10^{-4}$ and $\sigma_B = -10^{-4}$ (bottom). Lighter lines represent the first and last decile of the data.

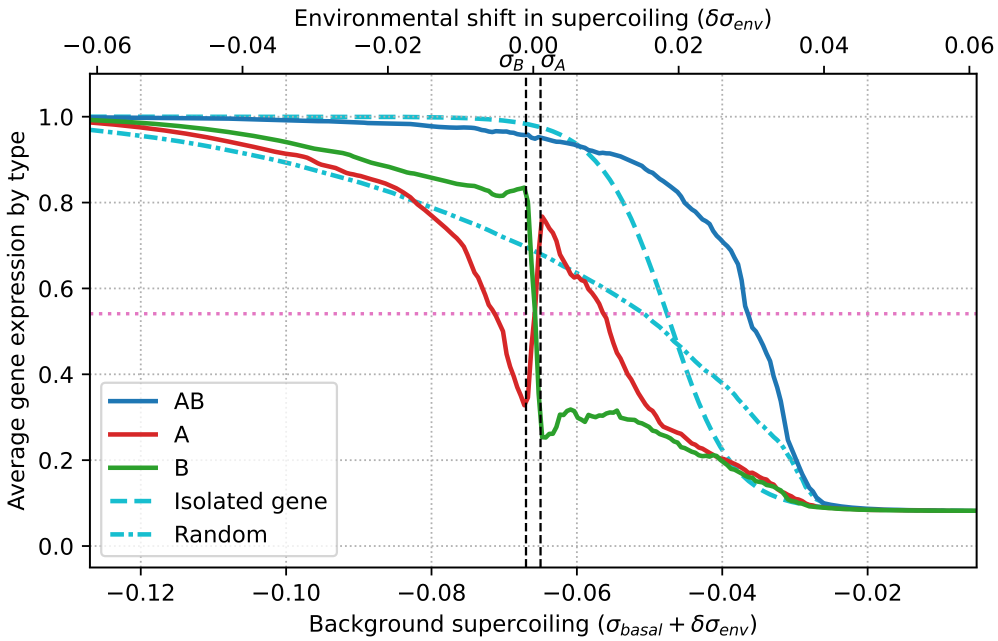

Figure C: Average gene expression as a function of background supercoiling, with environmental supercoiling shifts $\sigma_A = 10^{-3}$ and $\sigma_B = -10^{-3}$.

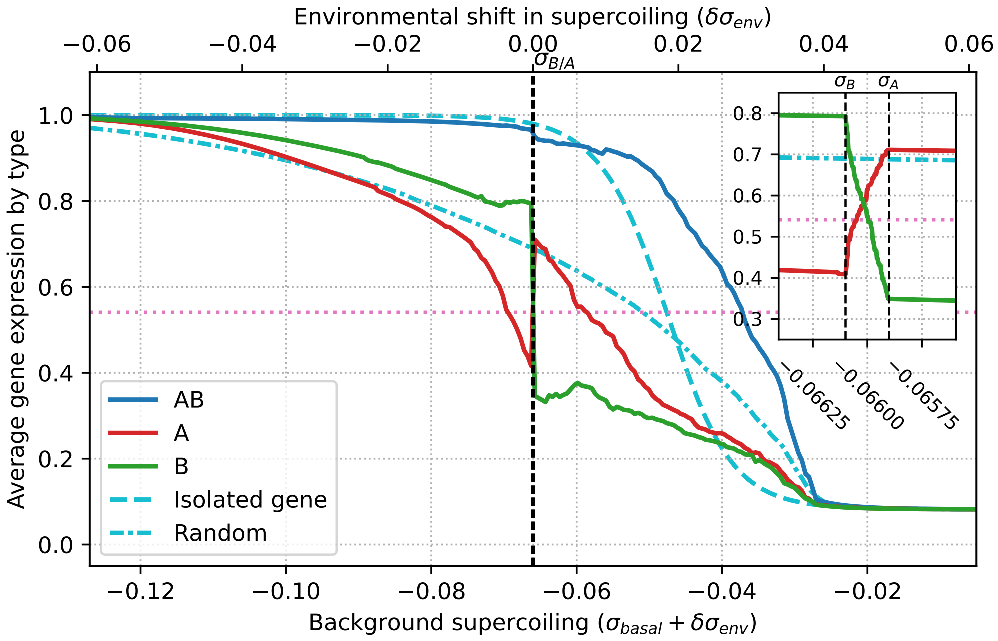

Figure D: Average gene expression as a function of background supercoiling, with environmental supercoiling shifts $\sigma_A = 10^{-4}$ and $\sigma_B = -10^{-4}$. The inset at the top right of the figure shows a 150x zoom on supercoiling shift values near zero.

Smaller Gene Interaction Distance

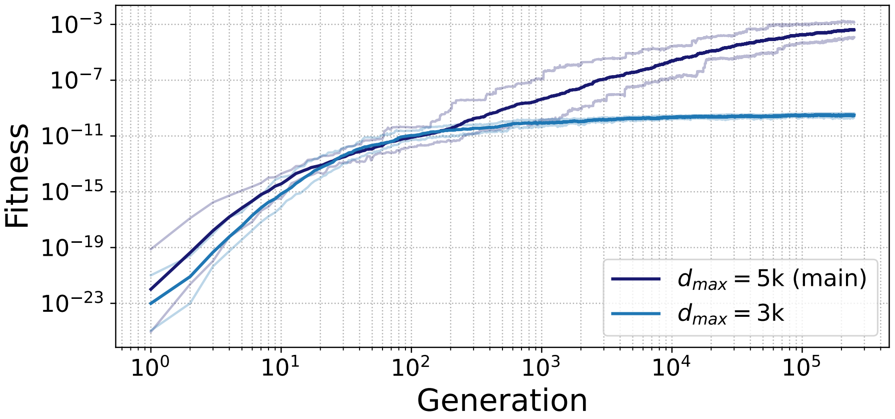

Figure E: Average fitness during evolution with different gene interaction distances: $d_{max} = 5{,}000$ bp (main run), $d_{max} = 3{,}000$ bp. Lighter lines represent the first and last decile of the data.

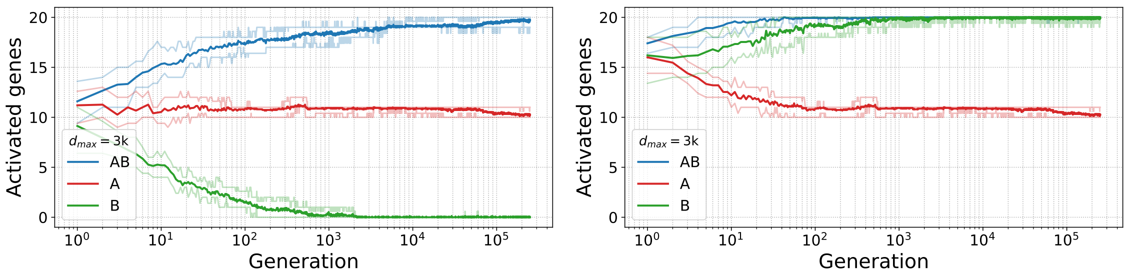

Figure F: Evolution of the number of activated genes in environment A (left) and environment B (right), with a gene interaction distance of $d_{max} = 3{,}000$ bp. Lighter lines represent the first and last decile of the data.

Population and Mutation Rate Rescaling

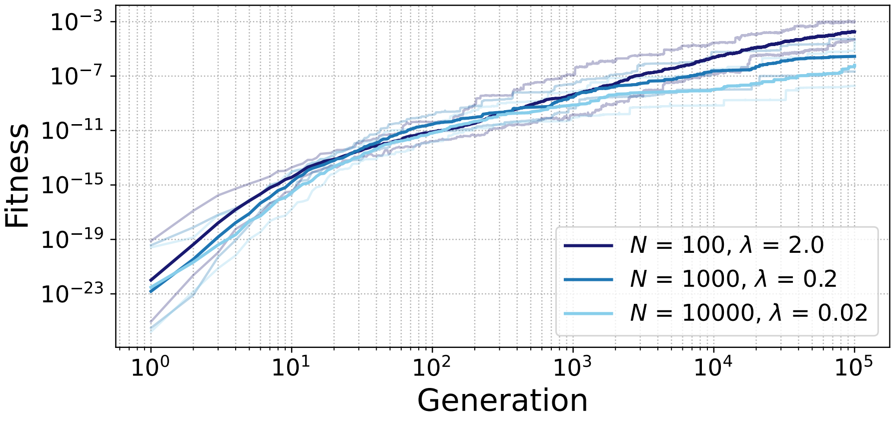

Figure G: Average fitness during evolution with constant $N\lambda$ but different N and λ values: $N = 100$, $\lambda = 2.0$ (main run); $N = 1{,}000$, $\lambda = 0.2$; and $N = 10{,}000$, $\lambda = 0.02$. Note that these simulations ran for 100,000 generations only due to computational constraints. Lighter lines represent the first and last decile of the data.

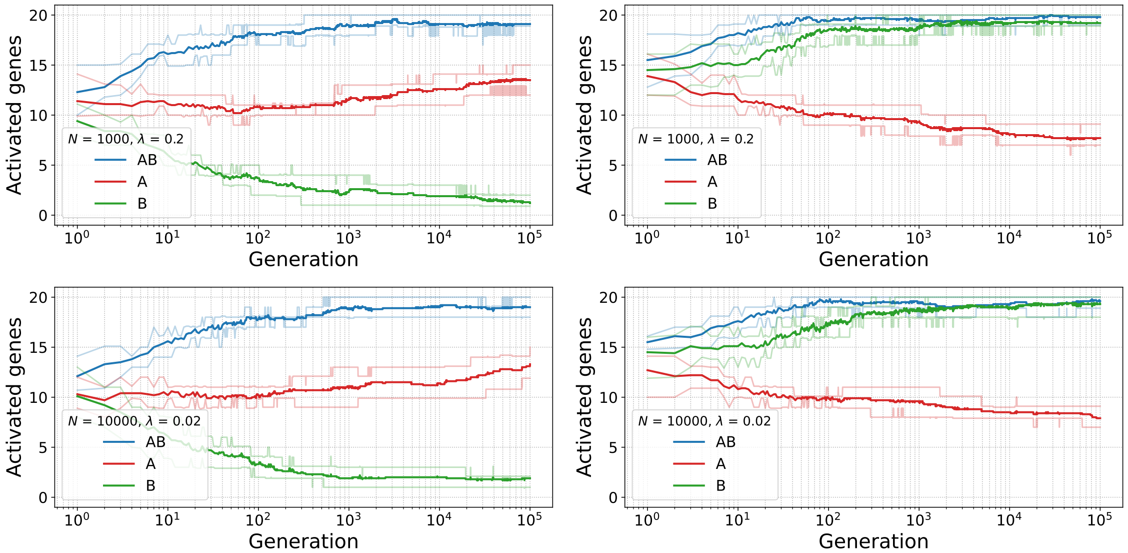

Figure H: Evolution of the number of activated genes in environment A (left) and environment B (right), with constant $N\lambda$ but different N and λ values: $N = 1{,}000$, $\lambda = 0.2$ (top); and $N = 10{,}000$, $\lambda = 0.02$ (bottom). Note that these simulations ran for 100,000 generations only due to computational constraints. Lighter lines represent the first and last decile of the data.
