# Supplementary material for: Emergence of supercoiling-mediated regulatory networks through the evolution of bacterial chromosome organization
Source: PLoS Comput Biol. 2025 Sep 29;21(9):e1013482. doi: 10.1371/journal.pcbi.1013482 (PMC12503335; doi:10.1371/journal.pcbi.1013482)
Supplement: Attachment — Submitted filename: reply plos cb round 1.pdf [file pcbi.1013482.s002.pdf]

Dear Editor,

We would like to thank the reviewers for their thorough reading of the manuscript, and the improvements that they are suggesting.

We reply in detail to their comments and suggestions below.

Reviewer 1

This study presents a computational model to analyze the role of DNA supercoiling in bacterial gene regulation and genome evolution. The manuscript is well-written and provides a thorough exploration of the transcription-supercoiling coupling and its implications for bacterial genome evolution. The use of computational modeling to study these interactions is a valuable contribution to the field, and the results potentially provide intriguing insights into the potential evolutionary consequences of supercoiling-mediated regulation.

A major issue with this paper is the lack of analysis on the relationship between the simulation results of proposed model and gene expression dynamics in actual cells. In bacterial cells, gene expression is regulated by transcription factors and other mechanisms. Additionally, during genome evolution, genes that benefit from correlated expression changes tend to form operons through structural mutations. While gene expression regulation via DNA supercoiling undoubtedly exists, its relative contribution compared to other regulatory mechanisms remains unclear. Therefore, it is difficult to assess how much the evolutionary properties identified in this study contribute to fitness in actual evolutionary processes when the simulation considers only DNA-supercoiling-based regulation.

The use of a simplified model incorporating minimal elements to analyze the relationship between gene expression and genome DNA structure in this study is understandable. However, for such a simplified model to be valuable, it should reveal novel insights into biological systems, even if it does not provide precise quantitative correspondence with real data. Unfortunately, the numerical experiments in this study only produce properties that are expected to emerge from the model's assumptions, i.e., the transcription-supercoiling coupling, making it difficult to recognize novelty in these findings.

To demonstrate the value of this study, the paper needs to establish some form of correspondence with experimental data. The most straightforward simulation result that could be compared with real data would be the relationship between gene orientation and changes in expression levels. For example, in E. coli, transcriptome data from various environmental conditions are available (Sastry, Nature Commun 10, 5536, 2019). If a correspondence could be found between expression changes, gene orientation, and the predicted genome structure as supercoiling in E. coli, the study would gain significant value. Given the abundance and accessibility of E. coli data, it would be a strong candidate for such validation. Another potential approach would be to analyze gene orientation and genome structure in organisms such as Mycoplasma, where regulatory factors are absent.

Answer: To us, the novel insight of our work is foremost that it showcases the ability for supercoiling-mediated interactions to serve as a substrate for genomic regulatory networks at scales ranging beyond two or three genes, rather than the quantitative prediction of the proportion of genes found in each possible relative orientation (and of the associated expression levels) in our model. This conclusion in itself makes it quite complex to compare gene orientation and expression levels, as we show that genes are interacting through a complex network mediated by several genes. Moreover, in dense genomes such as those considered here, it would be very difficult to isolate enough isolated pairs or triplets as we did it in (Meyer and Beslon, 2014) for instance. Hence, while we agree that a large-scale re-analysis of available experimental data would grant further empirical grounding to our hypothesis, we think that it would require new methodological developments that are clearly out of the scope of our manuscript. Our aim is that our theoretical study should help triggering such a research program in the near future: as the reviewer points out, supercoiling is not mentioned at all as a regulatory mechanism in Sastry (2009), but it is the case in the similar work of Yus (2019) on *M. pneumoniae* which we cite in the

manuscript, and our hope is indeed that researchers will seize this opportunity to expand the range of regulatory mechanisms that they consider.

Now, we of course agree with the reviewer that comparison of our results to real biological systems is of great importance to the relevance of any model and of its conclusions. To this end, we have introduced a new Table in the Discussion section of the manuscript (p. 26), aimed at giving an overview of how our hypothesis can be used in order to interpret a list of empirical observations that can be found in the recent literature. For each reference mentioned in the Table, we briefly describe the experimental method, the result, and how it can be seen under the prism of this work. We have also added several new experimental references in the revised manuscript, and we comment on these in the Introduction (line 131-136, p. 5; lines 152-164, p. 6) and Discussion as well (lines 579-581 and 583-587, p. 27; lines 642-647, p. 29).

Reviewer 2

This interesting manuscript focuses on the evolution of genome coiling and how gene transcription is affected by genome coiling, which in term can affect genome fitness. The model is well presented and reasonable to follow, though somewhat suffers from odd choices in model parameters such as tiny population size and massive mutation kernel.

Overall the model is well explored and thoroughly investigated for network size, knockouts, and global structure. While there are minor comments below, the major comment is that the biological relevancy appears low- there are a few bacteria that have evolved increased genome supercoiling discussed in the introduction, but even in these examples, protein changes, not inversion drove this genome evolution. It is unclear is any example exists of a gene inversion clearly increasing or decreasing fitness simply through expression effects on super coiling. As such, the mechanism of selection and evolution, while an interesting thought experiment, may have such weak selection coefficients that it is not observed. Is there an example of any gene, in any organism, where an inversion has a measurable effect in any environment on fitness?

Answer: We thank the reviewer for suggesting to investigate further the fitness effect of inversions in real bacteria. Some experimental work in *E. coli* (Fitzgerald 2021) and *S. enterica* (Waters 2022) has precisely shown that, with the advent of long-read sequencing, genomic inversions can now readily be found in populations of these bacteria, and that these inversions can have both deleterious or sometimes positive fitness effects. While the effect of these inversions on gene expression levels can be partially attributed to factors we do not consider in our model, such as dosage effects due to distance to the origin of replication and transcription-replication conflicts, it seems plausible to us that differential expression due to supercoiling-related feedback could also play a role in the fitness effects of inversions, as the highest difference is observed at inversion boundaries. In order to reinforce the empirical grounding of our hypothesis, we have introduced a new Table in the Discussion (p. 26), which gathers a list of experimental results that we interpret in the light of the two-way interaction of the transcription-supercoiling coupling with genome organization, and includes the two references mentioned above.

Minor comments: 1. Figure 1 would benefit from showing a random individual at the beginning of the experiment, as well as gene transcription levels as another ring.

Answer: We think that this would be redundant with Figure 12 (p. 31), which precisely shows an individual with randomly distributed genes. In that figure, the individual has fewer genes than in the main experiment to make it easier to describe in detail, but we could instead present an individual with the same genomic parameters as in the main experiment. We also added a reference to Figure 12 in the description of the model at the beginning of the Results section (lines 206-207 p. 7), as that figure was not referenced outside the Methods section.

2. The authors should explain their choice of a population size of 100 individuals and λ=2 inversions per replication event, as well as how their results would be different if population size of much larger and a much smaller mutation kernel were used instead. Given the regime for random selection into evolutionary dynamics, if population sizes of bacteria were huge and selection coefficients of rare inversions were smaller, would the same results be observed?

Answer: We agree with the reviewer that, in our model, the population size N and the mutation rate λ are very different than those observed in real bacterial populations. We have expanded the Methods section of the manuscript to explain this modeling choice in detail (new subsection 5.4, lines 844-869 p. 36-37). In essence, this is a classical problem of computational models of evolution, for which the usual solution is to reduce N (the major driver of computation time) while keeping the product $N\lambda$, representing the expected number of mutations coming in to the population at every generation, constant. In order to ensure that this rescaling does not affect our results, we conducted additional simulations with higher N and correspondingly lower λ (by factors of 10 and 100 respectively). These additional simulations gave us qualitatively similar results, which we have added to the Supplementary Material for completeness (Supplementary Figures S7 and S8, p. 5).

3. Figures 2 and 3- the first and last decile of the data's unusual to display and interpret. Why not display a standard deviation?

Answer: We decided to represent the first and last decile for two main reasons: first, showing deciles discards outliers (we have 30 data points at each time step). Second, contrary to standard deviation, deciles are not symmetric above and below the median, and therefore better represent the dispersion of the data, especially in the case of proportions which cannot by construction go below 0 or above 1 (such as Figure 3, p. 8). In order to remain consistent between Figures, we thus chose to use deciles everywhere. If the reviewer thinks that this choice makes it harder to analyse the figures, we would nonetheless be open to reconsidering showing the standard deviation instead.

4. Figure 4 is awesome.

Answer: Thank you!

Reviewer 3

In this manuscript, the authors present an analysis coupling a model for DNA supercoiling effects on bacterial gene transcription to evolutionary simulations to investigate whether supercoiling effects and the associated transcriptional effects can impact the organization of bacterial genomes adapting to two different environments with different global supercoiling levels and gene expression requirements, under the assumption that only supercoiling affects transcription (i.e. there are no transcription factors) and using one mutational operator: gene inversion. Their results show that the artificial bacterial genomes can evolve a genome organization in which genes are ordered and oriented so that their expression levels in the two environments (mostly) match the target phenotypes. From this, the authors conclude that DNA supercoiling can help shape both gene regulation and genome organization during evolution. The manuscript is very clearly written and the analyses performed are well-described. I liked reading the manuscript, but I do have a number of more conceptual questions on the analyses performed:

- A major concern I have is that the results presented may be evident given the model used. Given a model with a set of hard-coded rules, e.g. expression of divergently oriented genes induces negative supercoiling and negative supercoiling induces expression (positive feedback loop), only one influence on gene expression (supercoiling) and only one evolutionary operator (gene inversion), it is rather evident to me that evolution will use the inversion operator to position two AB genes that need to be always expressed (i.e. in environments A and B) in a divergent orientation. Even if the supercoiling-transcription model and the rule set used are true, the recovery of divergent AB gene pairs may be less evident if other regulatory influences (e.g. TFs) and other mutational operators (e.g. mutations in promoter regions) were taken into account. At the very least, potential modeling biases should be addressed in the discussion.

Answer: We agree with the reviewer that the model we present in this work is an ad-hoc model: its conclusions should not be interpreted as quantitative predictions on, for instance, the relative amounts of genes expected to be found in a particular syntax. While, as the reviewer points out, we describe in detail the local structures that evolve in the model, we believe that the key takeaway from the model is rather the evolution of large-scale gene regulatory networks based on supercoiling. We think that this work is particularly relevant in the context of the dense bacterial genomes, in which genes might interact with several close-by genes and not only their direct neighbors, in which supercoiling might play

a comparatively larger role. We also agree that introducing further complexity into the model would make it closer to biological reality, and could help answer additional questions (such as disentangling readthrough from supercoiling in operons), but we do not think that it would affect the main takeaway that is the evolution of those large-scale regulatory networks. We comment on those aspects in the Discussion (l. 609-640, p. 28-29).

- Why are the genomes adapted to two different environments simultaneously instead of one ? It is mentioned at some point that these are two environments that the bacteria may encounter during their life cycle, but it would be helpful to have concrete examples, in particular for obligate endosymbionts with low numbers of transcription factors such as B. aphidicola, for which supercoiling-based transcriptional regulation may be most relevant.

Answer: The reviewer is right to point out that this aspect of the model can seem rather ad-hoc. The reason for which we chose to model several environments is twofold. First, we were interested in studying how a different environment can mechanistically trigger changes in gene expression, and whether genome organization itself can provide sufficient expressiveness for such behaviors to evolve. In line with our hypothesis, genome-scale supercoiling variations have been measured in pathogenic bacteria such as *D. dadantii* (which must detect when it has found a substrate on which to activate pectinolytic genes) and *S. enterica* (which must detect when it has invaded epithelial cells); for *M. pneumoniae*, experimental work such as Yus (2019) has also used several environments to study gene regulatory networks, and this approach could doubtlessly be used to study *B. aphidicola* as well. Using several environments in our model therefore matches the experimental approach used in the *in vivo* study of regulatory networks. Our simulations show that, in the context of our model, a sensitivity of individual genes to supercoiling indeed provides a sufficient mechanism to evolve such regulation thanks to large-scale regulatory networks, which is the main takeaway of our work. Second, from a purely modeling perspective, using several environments and several gene types makes for a richer model to study than a single environment and a simple on/off target (although intermediate models could of course be envisioned). We show that, in the system with two environments, complex regulation can evolve: while we have not tested this experimentally, there is no reason to think that such regulation would not evolve in the simpler system with a single environment.

- The authors state that different models of transcription-supercoiling coupling produce contrasting results. Yet they use only one model, the one of El Houdaigui et al., 2019, in their simulations. Could the results qualitatively change when another model is used, or, the other way around, could matching the evolved genome organizational characteristics of different models with real genome characteristics support some model and rule out others ? E.g., are motifs observed in the simulation results, such as divergent pairs of always-on genes and convergent gene pairs with toggle switch-like behavior, also observed in reality ? More links to reality would definitely help to increase confidence in the biological relevance of the simulation results.

Answer: This question links to the first point: our model purposefully sets aside elements that are known to have an impact on supercoiling-mediated interactions, such as transcriptional readthrough and relaxation of positive/negative supercoiling constraints by topoisomerases, for example. While some of the experimental work on two-gene systems that we cite, like Boulas (2023) or new work such as Sarica (2025) focuses on matching quantitative predictions to experimental data and therefore needs to integrate all aspects to their model, the main takeaway of our work is again the prediction of the existence of complex regulatory networks at a larger scale than the one- or two-gene systems currently under empirical examination. Nonetheless, we agree with the reviewer that the manuscript would benefit from a closer comparison of our results with wetlab experimental results. In accordance with the this observation, we have emphasized this aspect further by integrating a Table of relevant experimental results (p. 26), describing in each case the experimental setup, and interpreting the results in the light of our work, which includes the two references mentioned above. We have also expanded the discussion of this aspect in the Conclusion (lines 661-668 p. 29).

Finally, as we point out in the Discussion (l. 609-640 p. 28-29), experimenting with different models of supercoiling-interaction between neighboring genes would undoubtedly strengthen confidence in the validity of the results, but we think that it is a necessary first step to understand well results from

first principles (the twin-domain model and its effects beyond two or three genes) before increasing the complexity of the model.

- The authors consider a gene on/off if its expression is above/below the expression threshold $e_{1/2}$, which is the average between minimum and maximum expression. However, the actual evolutionary optima are minimum expression (e^{-m}) or maximum expression (1). Judging from Figure 4, the evolved populations are rather far away from these optima after 1 million generations of evolution. How does this relate to the power of supercoiling to influence gene expression, what would e.g. be needed to really shut off a gene (and is it even possible ?).

Answer: In the context of the model, the answer to this comment depends foremost on the way that we compute fitness: it depends on the squared difference between the *mean expression level* per gene type and the expected expression, rather than the *sum over all genes* of the squared differences between observed and expected expression. This means that, for a given mean expression of genes of a certain type, it is more favorable to have homogeneous expression between genes of that type rather than more dispersed expression (with some genes closer to full inhibition). We have experimented with both methods for computing fitness: both approaches give very similar evolutionary trajectories, and we found the first one easier to work with. Second, the answer to this question also depends on the density of the genomes used in the experiment: genes are packed with little intergenic space, forcing multiple genes to interact at once through supercoiling. In an experiment where the quantity of intergenic space is higher, it is however quite possible to obtain full inhibition of genes in the model (see Section 6.2 of the PhD thesis of Grohens (2022) at https://hal.science/tel-04146510v1)).

Minor comments: - σ_0 on line 704 is defined as the level of supercoiling at which the opening free energy is at half its maximum level. Is this the same thing as the parameter σ_{opt} defined in Table 1 as the promoter opening threshold ?

Answer: We thank the reviewer for pointing this out: σ_0 on line 704 of the original manuscript indeed refers to what is termed σ_{opt} in the Table of parameters (p. 36). We have renamed this parameter $\sigma_{1/2}$ for clarity, and modified the main text of the Methods (lines 751-753 p. 33) and the Table of parameters accordingly.

- Parameter ε in equation 3 should be defined, it is linked in Table 1 to 'crossover width' but it's unclear what this means.

Answer: In this context, ε represents the sensitivity of promoter opening free energy to supercoiling, and is proportional to the derivative of the sigmoid curve $U_i(\sigma_i)$ at its inflexion point $\sigma_{1/2}$. We have clarified the description of this parameter both in the main text of the Methods (lines 751-753 p. 33) and in the Table of parameters (p. 36).

- The authors state on line 545 in the discussion that 'the distribution of gene triplets in evolved genomes is not uniform, but is enriched in configurations that are theoretically predicted to be beneficial in the model.'. I don't see a lot of solid theoretical predictions in the text. E.g. the authors state on line 359 that 'Triplet (2) shows how an AB gene in a strongly-expressed divergent ←AB — AB→ pair can inhibit a B gene, and the B gene of the triplet can also be seen as reinforcing the expression of the divergent ←AB — AB→ pair in environment B.', but reinforcing this divergent pair would lead to further inhibition of the B gene in the environment B where it is supposed to be expressed ? Couldn't it be that the enrichment of some triplets is just a consequence of the enrichment of some of the underlying pairs such as ←AB — AB→ ?

Answer: We agree with the reviewer's point that we have not provided a quantitative measure of whether some triplets are more frequent than expected, given the pair frequencies that we observe. This question is harder than it might look at first sight, because one would need to compare observed triplet frequencies to random triplet frequencies given an underlying distribution of pair frequencies obtained from evolved genomes. The problem is that this underlying distribution is not uniform; and, for example, reshuffling genes along the genome in order to sample the triplet distribution would break the underlying pair distribution and give a meaningless result. As such, we have rephrased the corresponding text in the Discussion to clearly state that our interpretation of the triplet frequency data is observational only (lines 583-587 p. 27).

- Figure 3: why are there already more activated AB and A genes than B genes in generation 1 of the adaptation to environment A, and more AB and B genes than A genes in generation 1 of the adaptation to environment B ? Is generation 1 the start point or is this the population after 1 generation of evolution (as I suspect) ? In the latter case, the horizontal trajectories before generation 1 do not help interpretation.

Answer: The reviewer is right: due to the logarithmic scale, generation 0 (the first population before evolution starts) is not plotted properly in Figure 3 and the corresponding Supplementary Figures. We have redrawn Figure 3 (p. 10) and Supplementary Figures S2, S6, S8 (Supp. p. 2, 4, 5) to omit this generation, in order to be consistent with the corresponding fitness plots.
